# Objects and Methods of Medication

**Published:** 1883-01

**Authors:** 


### THE OBJECTS AND METHODS OF MEDICATION.

IT IS generally conceded that medication is justifiable, only when there is reasonable hope that the benefits to be expected are greater than the injury that strong medicines often inflict. The object may be to remove costiveness by the use of some simple substance, as salts ; or to relieve the stomach of its contents by means of remedies as simple as mustard or common table stalt. Strictly speaking, this is not medication ; it is relieving the system of what oppresses it by methods that are simply mechanical. But medicines are those substances that produce changes in the system through their action on the general circulation. There are remedies that have a specific action on certain diseases, when properly administered ; they enter the circulation and destroy disease in its strong-hold. At the head of this list of alterative medicines stands mercury and the iodides. Quinine will prevent or cure intermittent fever ; and opium will relieve pains. It is of less importance to the physician to know the silent methods by which remedies produce their impressions, than to know how to apply them most judiciously and safely.

It would seem that when it becomes necessary to use drugs to combat diseases, it is desirable to get them to the point required as soon as possible and before the malady has time to make much head-way. Yet, strange to say, the road usually taken is the longest; it is that via the mouth to the stomach, where the medicines are mingled with its contents, thus undergoing chemical changes and dilution by contact with the gastric fluids, and then they must pass through the portal circulation before the active principle that remains can reach the general circulation; this often requires hours, while the remedies if applied to a mucous surface, or hypodermically, would enter the circulation in a few minutes.

Hypodermic medication is hazardous when morphine is used, probably because its full power is quickly exerted. All things considered, we believe the best way to administer most remedies is by SUPPOSITORIES. We in this way have no waste, the medicines being entirely absorbed by the mucous membrane in contact with it; the effect is not produced so rapidly as by hypodermic injections, nor so slowly and wastefully as by the stomach; still it must be remembered that one quarter grain of morphine or five grains of quinine will have as much effect when applied in suppositories as double these quantities taken by the mouth. In cases where the stomach will not tolerate food, life may be sustained indefinitely, by using HOLLOW SUPPOSITORIES filled with food extracts.

